# E-Cigarettes: A Review of New Trends in Cannabis Use

**DOI:** 10.3390/ijerph120809988

**Published:** 2015-08-21

**Authors:** Christian Giroud, Mariangela de Cesare, Aurélie Berthet, Vincent Varlet, Nicolas Concha-Lozano, Bernard Favrat

**Affiliations:** 1 Forensic Toxicology and Chemistry Unit, University Center of Legal Medicine (CURML), CH-1000 Lausanne 25, Switzerland; E-Mail: vincent.varlet@chuv.ch; 2 Department of Community Medicine and Health (DUMSC), Rue du Bugnon 44, CH-1011 Lausanne, Switzerland; E-Mails: aurelie.berthet@hospvd.ch (A.B.); nicolas.concha-lozano@chuv.ch (N.C.-L.); bernard.favrat@chuv.ch (B.F.); 3 Lausanne University Hospital (CHUV), Rue du Bugnon 46, CH-1011, Lausanne, Switzerland; 4 Unità di Medicina e Psicologia del Traffico, via Trevano 4, Casella postale 4044, CH-6904 Lugano, Switzerland; E-Mail: mariangela.decesare@umpt.ch; 5 Unit of Traffic Medicine and Psychology, CURML, CH-1005 Lausanne, Switzerland; 6 Institute for Work and Health (IST), Route de la Corniche 2, CH-1066 Epalinges - Lausanne, University of Lausanne and Geneva, Switzerland; 7 Center of General Medicine, Department of Ambulatory Care and Community Medicine (PMU), University of Lausanne, Rue du Bugnon 44, CH-1011 Lausanne, Switzerland

**Keywords:** cannabis, vaping, electronic cigarette, adolescence

## Abstract

The emergence of electronic cigarettes (e-cigs) has given cannabis smokers a new method of inhaling cannabinoids. E-cigs differ from traditional marijuana cigarettes in several respects. First, it is assumed that vaporizing cannabinoids at lower temperatures is safer because it produces smaller amounts of toxic substances than the hot combustion of a marijuana cigarette. Recreational cannabis users can discretely “vape” deodorized cannabis extracts with minimal annoyance to the people around them and less chance of detection. There are nevertheless several drawbacks worth mentioning: although manufacturing commercial (or homemade) cannabinoid-enriched electronic liquids (e-liquids) requires lengthy, complex processing, some are readily on the Internet despite their lack of quality control, expiry date, and conditions of preservation and, above all, any toxicological and clinical assessment. Besides these safety problems, the regulatory situation surrounding e-liquids is often unclear. More simply ground cannabis flowering heads or concentrated, oily THC extracts (such as butane honey oil or BHO) can be vaped in specially designed, pen-sized marijuana vaporizers. Analysis of a commercial e-liquid rich in cannabidiol showed that it contained a smaller dose of active ingredient than advertised; testing our laboratory-made, purified BHO, however, confirmed that it could be vaped in an e-cig to deliver a psychoactive dose of THC. The health consequences specific to vaping these cannabis preparations remain largely unknown and speculative due to the absence of comprehensive, robust scientific studies. The most significant health concerns involve the vaping of cannabinoids by children and teenagers. E-cigs could provide an alternative gateway to cannabis use for young people. Furthermore, vaping cannabinoids could lead to environmental and passive contamination.

## 1. Introduction

### 1.1. Context

There are countless ways to consume cannabis. Means of administration are only limited by the imagination of manufacturers, resellers, and users, and by the technology of the devices used for administration. However, inhalation remains the most popular method of cannabis consumption. Instead of traditional marijuana cigarettes (*i.e.*, cannabis joints), the electronic cigarette (e-cigs) has emerged as the best option for those who are fond of new technological gadgets [1]. It is claimed that e-cig aerosol contains fewer harmful chemicals than ordinary tobacco cigarettes and possibly than regular marijuana cigarettes [2,3]. Gostin’s paper [4] on nicotine vaping and youth raises new issues about e-cigs and their potential misuse for consuming cannabis and other psychoactive drugs.

### 1.2. E-cigarette Hardware

**Figure 1:**
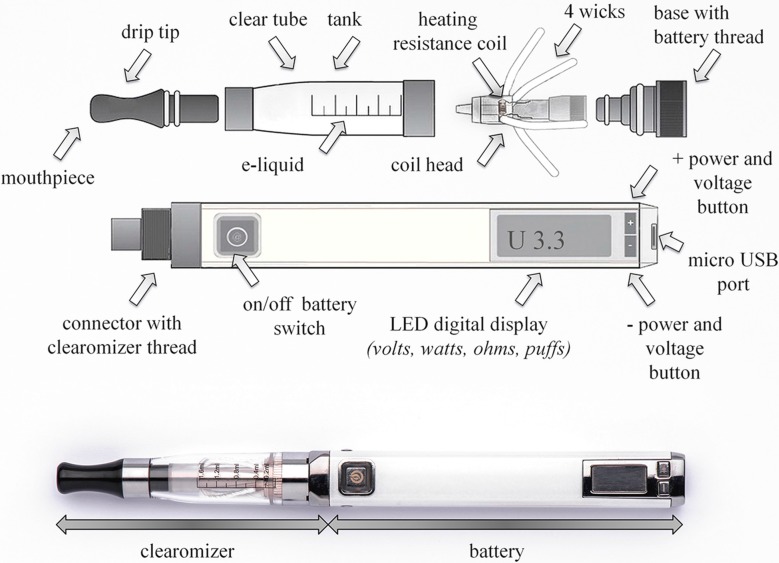
Microprocessor-controlled, variable-voltage/wattage, personal electronic-cigarette with LED digital display (volts or watts, puff count, ohmic resistance), equipped with a transparent clearomizer and changeable dual-coil head. Vaping cannabis requires an e-cigarette model of this type, offering a wide range of settings (power, voltage adjustments) and the option of using different resistance coils. The presence, number, and positioning of wicks are also important.

Vaping nicotine using e-cigs differs from smoking regular tobacco cigarettes in many ways. Early, first-generation e-cigs were usually designed to simulate smoking regular tobacco cigarettes; they were low-tech vaporizers with a limited number of settings [1]. Second-generation e-cigs and third-generation modified e-cigs (mods) use more advanced technology; they have atomizers (*i.e.*, heating coils that convert e-liquids into vapor) which improve nicotine dispersal and house high capacity batteries [5]. Cartomizers are similar in design to atomizers; their main difference is a synthetic filler material wrapped around the heating coil. Clearomizers are now very widespread and similar to cartomizers, however they include a clear tank of a larger volume and no filler material; additionally they have a disposable head containing the coil(s) and wicks. Figure 1 shows a diagram and picture of a typical e-cig equipped with a clearomizer. The coils can be fitted to the top of the tank (as in Figure 1), with long wicks hanging down into the tank or at the bottom of the tank with short wicks. E-cigs with advanced technology that includes a large number of settings and the possibility of replacing some critical parts are best suited to cannabis vaping.

Because they are closer to the mouthpiece than bottom coils, top coils deliver warmer vapors than bottom coils. More recently, wickless clearomizers have been marketed. These use a central cotton core to absorb the e-liquid and feed it to the heating coil. Several holes on the inner tube allow the e-liquid to flow easily through the clearomizer. These advanced technological devices may also contain a built-in electronic chip to adjust the voltage and power of the heating element, and an ohmmeter to measure the electrical resistance of the coil. Some clearomizers can be disassembled to remove and replace the atomizer and coil(s). A coil with another resistance can be fitted. At constant voltage, the lower the atomizer’s resistance, the greater the amount of aerosol generated. Each level of resistance also requires a certain voltage/power combination for optimal performance and vapor production. For a specific coil and resistance, the gradual increase in voltage progressively produces no, small, significant and optimal amounts of warm vapors, or even hot smokes. Numerous websites and user forums report that, under optimal operating conditions (e.g., 3.7 V tension battery and 1.5–1.8 ohm resistance coil), power loads ranging between 8 and 9 watts are ideal for procuring large amounts of vapor, very pleasant sensations, and the typical throat hit that many users highly appreciate. This electric power setting was also considered as optimal and was selected by Farsalinos *et al.* (2014) for comparing the efficiency of nicotine delivery between first- and latest-generation e-cigs [6].

**Figure 2:**
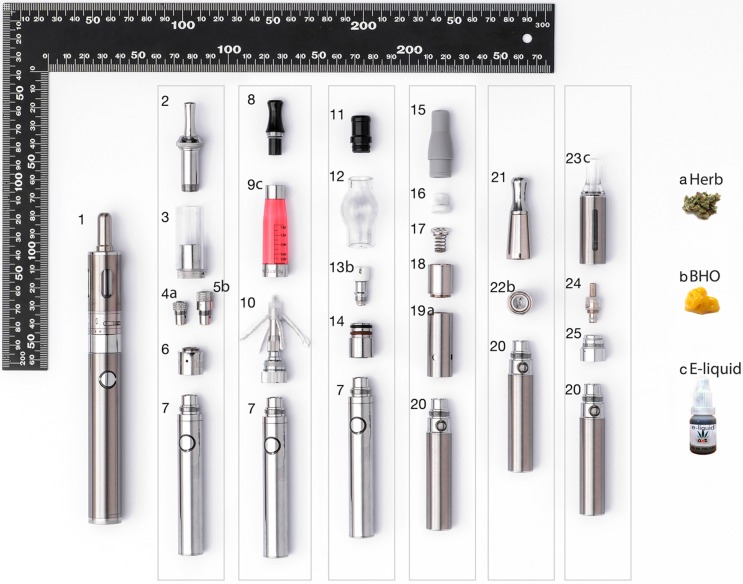
Some models of e-cigs for vaping (**a**) ground marijuana head tops; (**b**) cannabis wax (butane honey oil concentrate (BHO)), or (**c**) cannabis e-liquid. The letters a, b, or c after the numbers indicate the parts of e-cigs used for herb, BHO, or e-liquid vaping, respectively. 1. Mega electronic cigarette (e-cig) with dual-coil clearomizer, adjustable airflow control ring, changeable resistance coils and high capacity, variable voltage battery. 2–7. Dry herb and wax vaporizer: 2. Spring-loaded mouthpiece; 3. Vaporizer chamber; 4. Dry herb coil head; 5. Wax coil head; 6. Detachable base with air holes; 7. Medium capacity battery. 8–10. See-through clearomizer for e-liquids: 8. Clearomizer tip; 9. Clearomizer tube; 10. Clearomizer coil and four-wick head with base assembly. 11–14. Glass-globe atomizer wax tank: 11. Drip tip; 12. Glass globe; 13. Ceramic heating chamber and coil; 14. Metal core. 15–19. Dry herb atomizer: 15. Soft drip tip (mouthpiece); 16. Ceramic screen; 17. Metal screen and spring; 18. Screens connector; 19. Ceramic chamber and battery connector. 20. Medium size battery. 21–22. Wax coil head atomizer: 21. Metal mouthpiece and wax coil chamber connector; 22. Wax coil ceramic chamber and battery connector. 23–25. Clearomizer for e-liquids: 23. Shell (mouthpiece attached to metal tank with viewing window); 24. Bottom head changeable coil assembly; 25. Battery base connector. a. Marijuana head tops, b. Cannabis Butane Honey Oil concentrate (BHO), c. Cannabis e-liquid (mixture of cannabinoid concentrates (cannabis wax), propylene glycol and glycerol).

Developing on these features, several types of e-cigs were designed and adapted so as to be able to vape dry herbs, oil concentrates, or cannabis-based e-liquids as shown in Figure 2. The ability to regulate the evaporation temperature significantly raises the attractiveness of using e-cigs for vaping psychotropic drugs, such as (−)-trans-Δ^9^-tetrahydrocannabinol (THC), the main psychoactive cannabinoid in marijuana. In addition to THC, supposed treatments for various pathologies can now be bought from legal and illegal drug retailers, based on cannabinoids other than THC (e.g., cannabidiol, cannabigerol) [7,8], and could be administered by vaping through conventional e-cigs or very likely more efficiently with e-vaporizers. Recently, a special device was even patented to make e-cigs compatible for vaping nicotine [9], THC, tobacco, cannabidiol or alkaloids. Solowij *et al.* [10] reported optimized protocols for the delivery of cannabidiol, and combined cannabidiol and THC, by vaporization using a Volcano^®^ table vaporizer (Storz & Bickel, Tuttlingen, Germany). In parallel with this, innumerable Internet forums describe attempts and failures to manufacture hash oil mixtures using tinctures or vegetable glycerol/propylene glycol or, less frequently, polyethylene glycols (mixtures of PEG 200, 300 and 400) that can be vaped as e-liquids in e-cigs.

The fact to heat herb extracts at lower temperatures (140 °F –374 °F or 60 °C–190 °C) than combustion temperatures (1472 °F –1652 °F or 800 °C–900 °C) can still produce an inhalable vapor that can be vaped in e-cigs. Although the vapor thus contains smaller amounts of harmful pyrolyzed by-products, it still contains the psychoactive ingredients (e.g., nicotine, or cannabinoids from marijuana). Controversy continues surrounding the real or supposed benefits and risks of e-cigs that deliver nicotine vaporized from enriched e-liquids [5], particularly among youth [4], yet other issues also deserve attention.

To assess the different options available for vaping cannabinoid aerosols using e-cigs, a short literature review was carried out. This review is intended primarily for medical staff involved with cannabis-related health issues as this target audience is generally misinformed about the possible uses of e-cigs for vaping cannabis extracts, as well as how e-cigs work. Forensic scientists will also benefit from this review. The different types of e-liquids and the various manufacturing methods for preparing cannabis concentrates are described in order to improve understanding of the various ways of using e-cigs for marijuana inhalation. The review concludes with a discussion on the respective advantages and disadvantages of regular cannabis joints and e-cigs, including the underlying major health risks and safety hazards of vaping for youths, teenagers, and non-smokers. Some recommendations for mitigating public health risks are also proposed. Because of the paucity of the available scientific data regarding cannabis and vaping, we often had to rely on information about nicotine vaping, which has been more thoroughly examined for many of these aspects. For the same reason, the review took account of the very rich underground literature and presented some original experimental results from our laboratory on a cannabidiol-enriched e-liquid and on cannabis butane honey oil concentrate (BHO).

## 2. Literature Search

Systematic literature searches were conducted using the PubMed, Web of Science, Google Scholar, and Embase databases, using the following set of relevant search terms: (“electronic cigarette” OR “e-cigarette” OR “e-liquid”) AND (“cannabis” OR “marijuana”). The regular Google search engine and grey literature (e.g., ref. [11]) were also consulted. The literature search covered 2003 to July 2015; 2003 corresponds to the development of the first e-cigs by Hon Ilk, in China.

## 3. Vaporized Material

### 3.1. Context

A synthesis of the practical recipes and experiments reported on the Internet (in addition to our own experiments) shows that the simple, direct use of purified cannabis extracts in e-cigs is not easy because cannabinoids are poorly soluble in e-liquids. Indeed, used pure or in mixture with glycerol, the propylene glycol (PG, or propane-1,2-diol) that is commonly used in e-liquids has both hygroscopic and hydrophilic properties. Consequently, it is not miscible with mineral and vegetable oils and fats. In contrast, cannabinoid concentrates are very lipid soluble, but water insoluble. Furthermore, PG, glycerol, terpenoids, plant fats and waxes, cannabinoid concentrates and THC are very viscous substances. Accordingly, the manufacture of a homogeneous solution using these viscous liquids is a tedious task. Cannabinoid concentrates resemble more a thick, sticky, gummy resin than a liquid. We can infer from these facts that they do not mix readily with glycols, and even less so with glycerol. The addition of terpenoids (e.g., limonene) or lecithin has been advocated to help make cannabis oils miscible [12]. Adding flavors (e.g., menthol, tobacco, orange crush) with a fragrant aroma to e-liquids can mask the typical, strong odor of cannabis drugs and make it less detectable. Although glycerol and propylene glycol are not ideal solvents for dissolving cannabinoids, propylene glycol is an excellent emulsifier and can be used to make e-liquids, such as (BHO)-PG emulsions. To do this, BHO is dispersed in PG solution using a modular high-speed homogenizer drive-unit equipped with a dispersing shaft (such as an Ultra-Turrax blender, IKA, Germany). The main problem with emulsions is that they are not often fully stable, and after a length of time they tend to separate progressively into two phases [13]. The separated BHO droplets may also stick to various parts of the e-cig, such as the tank walls or the wicking elements. The inhomogeneity of e-liquid could be a further problem: a particulate phase, made up of fine debris of plant tissues and trichomes, can coexist with the emulsion. A significant part of the cannabinoids can be contained in the trichomes or adsorbed on cellular debris. These insoluble materials can damage or impede the proper functioning of the atomizer. Just as with tobacco smoke, this particulate phase can be harmful to the lungs. Most of these problems can be prevented by performing intensive purification of the BHO (winterization and fine filtration), diluting it appropriately in glycols, and avoiding the use of glycerol. Exceeding a 1:2 w/w ratio between the purified BHO and the e-liquid (neat PG or mixture of glycols) usually leads to a phase separation [14].

### 3.2. Manufacture of Marijuana Vape E-Liquids

**Figure 3:**
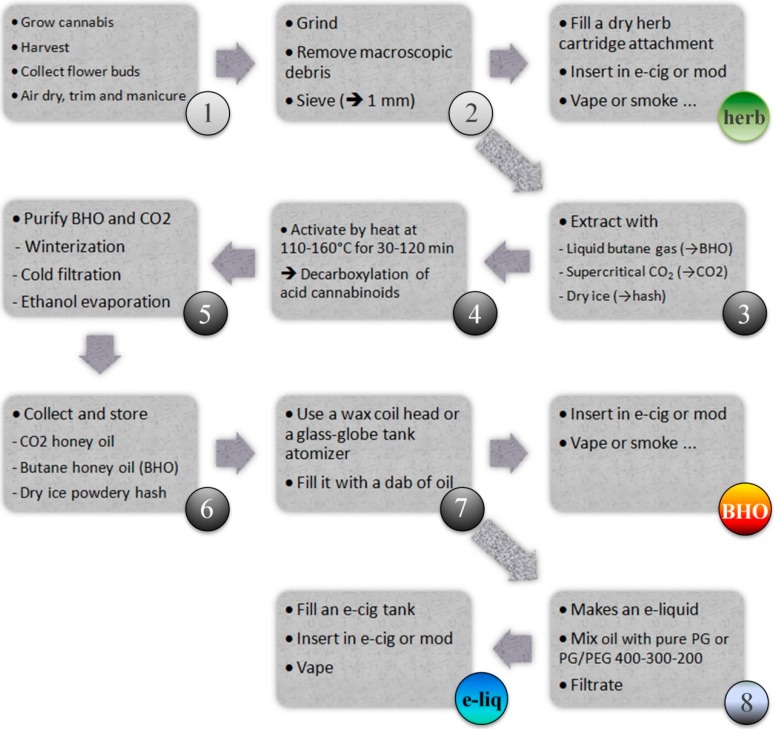
Manufacture of ground cannabis head buds, butane honey oil extract and cannabis e-liquid. 1–3. Processing cannabis heads and smoking them in a dry herb vaporizer; 4–9. Manufacture of cannabis oil concentrate and vaping with a wax vaporizer; 10–11. Making a cannabis e-liquid and vaping in a regular e-cigarette or e-mod.

The preparation of marijuana vape e-liquids requires several steps (see Figure 3): cannabinoids from dried and finely pulverized cannabis flower buds must be heat-activated, extracted, and purified. After harvesting and drying the raw mature plants, the flower tops are ground to fine particles. The milled fragments are then heated (range, 230 °F–310 °F or 110 °C –160 °C) to convert the inactive natural acid plant precursor, tetrahydrocannabinolic acid A (THC-A), into its neutral and psychoactive THC counterpart [15,16]. At the same time, the other acid cannabinoids are also converted to their neutral counterparts by heat-decarboxylation. Overheating must be avoided because it may result in a degradation of the THC and the formation of substantial amounts of cannabinol. Lower temperatures require longer decarboxylation times (>1 h), but they preserve most of the cannabinoids and the aromatic and volatile terpenoids, and prevent losses of THC by evaporation. However, this heat-decarboxylation step generates CO_2_ and an increase in pressure, creating a risk that the container might burst and rupture. To mitigate their degradation by oxidation and evaporation, the plant fragments must be thermally processed in a large, closed, robust container filled with inert gas (e.g., nitrogen, argon). This decarboxylation stage (see Figure 3, step 3) can take place before or after the extraction (step 3) and purification phase (step 5) of the plant’s cannabinoids. Besides using organic solvents (e.g., ethanol, isopropanol), some of the most popular extraction methods involve using dry ice (non-flammable solid pellets of frozen carbon dioxide, CO_2_), highly inflammable liquid gases such as butane, or low-viscosity supercritical (SC) fluids such as SC-CO_2_. These methods have several advantages: extraction is rapid and takes place at very low or moderate temperatures; after elution, filtration through a coarse cellulose filter (e.g., coffee filter or Whatman 595 filter paper), depressurization, and heating, CO_2_ or butane is vented to leave a crude cannabinoid-enriched fraction. Any remaining traces of extracting fluid are very easily eliminated by evaporation at moderate temperatures (e.g., 140 °F or 60 °C in a water bath). This soft extraction process preserves the chemical integrity of the cannabinoids. The main drawbacks are the risks of gas inhalation or explosion. The resulting waxy concentrates are named CO_2_ and BHO (butane honey oil). These relatively crude extracts can be further purified by ethanol solubilization, followed by slow, deep-freeze precipitation (for at least 2 days) and the out-filtration of unwanted waxes and triglycerides via a glass wool filter in a separating funnel. This process, known as winterization, is the classic bio-industry method to remove traces of waxes and high-melting glycerides from fats. Skipping this step may expose the consumer to a risk of lipid pneumopathy. Exogenous lipoid pneumonia [17] due to vaping of glycerol-based e-liquids may also occur [18]. Lastly, the ethanol phase and solvent residues are evaporated at 40 °C–60 °C, or in a tepid bain-marie, under a fume hood and dried in an evacuated bell jar or a vacuum chamber. The purified yellow, wax-like residue is kept in the dark in an airtight container before consumption. Even tiny amounts of these concentrates, which can be put in the small-volume containers of e-cigs and mods, contain sufficient amounts of THC to provide the desired, typical psychoactive effects of cannabis [19]. This purified concentrate can be also mixed and diluted with pure propylene glycol (PG) or with mixtures of polyethylene glycols and PG, as advocated by EJMIX (Liquidizer, Bloomsdays, USA) providers and users [20].

### 3.3. Feasibility and Efficiency of Cannabis E-Liquid Vaping Using Electronic Cigarettes

The rate at which a drug enters the brain largely determines its euphoric effects. Recreational cannabis users will, therefore, prefer to achieve peak blood concentrations of THC by choosing e-cigs able to deliver a significant dose in a minimum amount of time. To achieve these goals, two important parameters must be met. First, the maximum THC blood level must be reached in a reasonable number of puffs (e.g., fewer than a dozen). Second, the length of time required to reach this peak concentration must be as short as possible (e.g., a dozen of minutes). It is known that each puff consumes about 5 mg of e-liquid [21]. The maximum proportion of BHO in e-liquids is about one third, but good quality BHO (decarboxylated and purified) may contain 80% THC. Therefore, 5 mg of BHO e-liquid could contain 1.3 mg THC. Using a bioavailability of just 30% and a minimum THC psychoactive IV dose of 1.5 mg [22], a mere four puffs could deliver about 4 × 0.4 mg THC = 1.6 mg THC into general blood circulation—A high enough dose to produce a psychotropic effect.

### 3.4. Vaping without E-Liquid

To circumvent the poor solubility of cannabinoids, some e-cig and mod manufacturers propose dedicated equipment for vaping wax or solid plant material without the need for e-liquids. To this end, interchangeable coil heads for wax, glass-globe tank atomizers for smoking refined hash oil, and coil heads for smoking finely ground, dried cannabis buds are now available. Figure 2 shows several of these portable vaping devices. For instance, dry herbs can be vaped with vaporizer models 2-3-4-6 or 15-16-17-18-19. BHO is better inhaled using the glass-globe atomizer wax devices 11-12-13-14 fitted to any appropriate battery (e.g., 7 or 20). The efficiency of acid cannabinoid decarboxylation and vaporization should be quite good if the e-cig coil-head temperature is sufficiently high (about 356 °F or 180 °C). Another efficient method of releasing the carboxylic acid group is by placing a bowl of buds or the cannabis concentrate in the microwave for a few minutes or in a regular kitchen oven for a longer time (Figure 3). Advanced e-cigs or mods and portable advanced vaporizers, used to vape herbal marijuana and waxy concentrates, are derived from large, table vaporizers that are considered effective and medically sound cannabinoid delivery systems [23]. These more sophisticated devices have several advantages over traditional e-cigs. For example, they contain glass-lined ceramic bowls that regulate the temperature for an efficient vaporization of the blend inserted into the device. The temperature setting and range generally goes up to 430 °F (212 °C) and can be time-programmed. In traditional e-cigs, an overly high temperature and a prolonged contact of the heating coil with the concentrate must be avoided to prevent the combustion of the cannabis material and the formation of toxic pyrolytic by-products.

### 3.5. E-liquids Made with Synthetic Cannabinoids

Other recipes found on the Internet suggest substituting synthetic cannabinoids (e.g., JWH-018, APINACA and AB-FUBINACA) for THC [24]. Synthetic cannabinoids can be mixed into e-liquids and inhaled through a pen-sized vaporizer as liquid synthetic pot (liquid spice). Non-polar synthetic cannabinoids sprayed on aromatic herbs can be vaped directly and more efficiently with e-cigs equipped with dry herb coil heads than in e-cigs fitted with e-liquids.

### 3.6. Vaping Recreational Drugs with E-Cigarettes

In addition to cannabis, e-cigs can also be used to vape any type of psychoactive drugs, such as methamphetamine, cocaine, heroin, or bath salts (cathinones). Until now, information on the use of e-cigs for the vaporization of THC-enriched aerosols has come mainly from the Internet, the popular press, street healthcare workers, and user’s testimonials, but not from reliable scientific literature. For instance, dedicated magazines publish buyer’s guides to vape pens, reviewing dozens of e-devices able to vaporize wax or other hash-style concentrates, but also flower buds or even oil concentrates. Very recently, drug users have discovered a method of adapting e-cigs to vaporize a potent hallucinogen known as dimethyltryptamine or DMT [25]. Many personal experiences about vaping crystal methamphetamine are also reported on the Internet [26]. Similarly, the use of e-cigs as a means of administering numerous controlled substances is abundantly discussed on websites and forums.

## 4. Analytical Characterization of Selected E-Liquids

### 4.1. Context

Due to the paucity of available scientific data and in order to illustrate the preparation of cannabis e-liquids and BHO, our laboratory performed a series of assays on a commercially available cannabidiol e-liquid and a home-made BHO. Firstly, we analyzed the cannabidiol e-liquid using gas chromatography-mass spectrometry (GC-MS) to identify the main constituents present in the solution. Cannabinoids were then quantitated by means of high-pressure liquid-chromatography using reference standards. In a second phase, BHO was prepared and mixed with various mixtures of glycols in order to obtain the most homogenous and stable e-liquid possible. Pure BHO or BHO mixed with e-liquid were then analyzed for THC-A, THC, and cannabinol (CBN) content. The methods and results of these assays are detailed below.

### 4.2. Methods of Analysis

Cannabinoid standards were ordered from THC Pharm (Frankfurt, Germany) (THC-A, 1 mg/mL methanol; CBD-A, 1 mg/mL; cannabigerol, 1 mg/mL methanol), Lipomed (Arlesheim, Switzerland) (THC, 1 mg/mL methanol; cannabinol (CBN), 1 mg/mL methanol; cannabidiol (CBD), 1 mg/mL methanol), and Restek (cannabichromene, 1 mg/mL methanol). *N*-Methyl-*N*-trimethylsilyl trifluoroacetamide (MSTFA) was purchased from Macherey-Nagel (Oensingen, Switzerland). A capillary column (J & W DB-XLB 30 m × 250 μm × 0.25 μm film thickness) was obtained from Agilent Technologies (Basel, Switzerland). All solvents used to prepare e-liquids (polyethylene glycol 200, 300 and 400, propylene glycol and glycerol) were from Sigma Aldrich. Acetonitrile, methanol, ethanol, and formic acid were of analytical grade (Sigma-Aldrich). Different e-liquids and cannabis extracts were analyzed by GC-MS using an Agilent GC 6890N interfaced with a 5973 MSD, as follows: (a) butane honey oil (BHO) extract (35%) in a mixture of propylene glycol/polyethylene glycol 200, 300 and 400 e-liquids (EJMIX, Bloomsdays, USA); (b) e-liquids containing 2% cannabidiol (CBD) and 0.5% tetrahydrocannabinol (THC) from cannabis sativa bio extract mixed with propylene-glycol and glycerol in 1:8:1 w/w/w proportions (Cannaxtract, Switzerland), respectively, were diluted with methanol down to 10–100 μg THC/mL. A 10 μL aliquot was dried using an air-flow and silylated for 30 min at 80 °C in a 100 μL MSTFA/acetonitrile 1:1 v/v mixture. 1 μL was injected splitless at 270 °C onto a J&W DB-XLB column at a constant flow of 1.2 mL/min. The initial temperature was 70 °C. After 1 min, the temperature was ramped up by 15 °C/min to 200 °C, then by 10 °C/min to 300 °C. This temperature was maintained for 7 min and then increased by 30 °C/min to reach the final temperature of 320 °C. The total run time was 31 min. After a 3.5 min solvent delay, the MSD detector (set to EI mode) was switched on; ions were scanned between 10 and 400 atomic mass units (a.m.u.) for 7 min, then between 30 and 550 a.m.u. at a threshold of 20 mV. The MS and quadrupole temperatures were set at 230 °C and 150 °C, respectively. A PBM search was carried out using the MPW2011 and Nist2014 drug databases. Formal identification was obtained by comparing the retention time and mass spectrum of the silylated derivative with those of authentic samples.

Cannabinoids in e-liquids and BHO extracts were quantified using high-performance liquid chromatography (HPLC) using an Agilent 1100 Series system with diode array detection. Cannabinoids were separated by gradient elution on a Macherey-Nagel (Düren, Germany) CC 250/3 Nucleodur 100-5 C8 ec column, protected by a MN CC 8/3 Nucleodur 100-5 C8 ec precolumn. Reverse phase gradient elution was obtained by mixing two eluents: (A) methanol/water (1:1, v/v) and 25 mM formic acid; and (B) methanol and 25 mM formic acid. The column flow was 0.5 mL/min and the run time was 30 min. The initial proportion of eluent B was 40%. This proportion was increased linearly up to 100% over 25 min. The final eluent composition was maintained for 3 min. A linear calibration curve for THC-A, CBN, CBD, and CBD-A was obtained from 5 µg/mL to100 µg/mL. Samples were diluted to appropriate concentrations of cannabinoids ranging from µg/mL 20 to 100 µg/mL. 10 µL of diluted extract in solvent A were injected into the column. Cannabinoids were identified by comparing their retention time and UV spectrum with those of authentic samples and by performing a library search in the Pragst, Herzler, Herre, Erxleben, Rothe 2001/2007 UV-Vis database. UV spectra were registered from 195 nm to 300 nm with a range step of 2 nm. The library was completed with the spectra of all commercially available cannabinoids.

### 4.3. Analysis of a Commercially Available Cannabidiol E-Liquid

Several putative health benefits of the main non-psychoactive cannabinoid, cannabidiol, are currently under investigation. Cannabidiol is presently considered as a potential therapeutic drug because of its anti-inflammatory, neuroprotective, anti-psychotic, anxiolytic, anti-epileptic, and anti-cancer effects [27,28]. In this context, a few alternative and allopathic cannabidiol-based medicines are already available. For instance, cannabidiol is the unique active substance of Epidiolex^®^ (GW Pharmaceuticals, Cambridge, United Kingdom), a new therapeutic drug that has been evaluated as a potential treatment for childhood epilepsy in Dravet syndrome [29]. We recently purchased e-liquid (Cannaxtract eliquid) purported to contain 2% cannabidiol and 0.5% THC, via the Internet. This liquid can currently be sold in Switzerland due to the unclear legal framework, taking advantage of the argument that below the threshold concentration of 1% THC, the e-liquid is not a narcotic drug. This e-liquid’s real market appears to be at the crossroads between recreational drugs and alternative medicine; the preparation claims to make the user more peaceful, confident, aware, and mindful. Under examination, by eye and microscope, this new type of e-liquid revealed itself to be a translucent solution containing macroscopic and microscopic plant debris and hairs. Gas chromatography-mass spectrometry analysis revealed the presence of trimethylsilyl derivatives of propylene glycol and glycerol. Sesquiterpenes (caryophyllene), which are partly responsible for the typical scent of cannabis, as well as polyols (e.g., xylitol, mannitol) and sugars (fructose), were also detected. Trimethylsilyl derivatives of acid (cannabidiolic acid, cannabigerolic acid, cannabichromenic acid, cannabidivarinic acid) and neutral (cannabidiol, tetrahydrocannabinol, cannabinol) cannabinoids were also found. Cannabidiolic acid is the plant acid precursor of cannabidiol, whereas cannabidivarinic acid is the same as cannabidiolic acid, but with the side-chain shortened by two methylene bridges. Quantification of the main cannabinoids, using high performance liquid chromatography-diode array detection, revealed that most of the cannabinoids were present in amounts below (about 20% less) than those indicated on the e-liquid container. Analysis of another specimen from the same manufacturer but bought from another retailer revealed much lower concentrations of CBD and THC. Degradation during inappropriate and prolonged storage could account for these differences. It is very likely that the extract was heat-treated in order to decarboxylate the acid cannabinoids because only 10% of total cannabidiol (free and in acids) was recovered in the form of cannabidiolic acid. This hypothesis is supported by the non-detection of the monoterpenoid molecules typical of cannabis flower buds (e.g., myrcene, pinene); these volatile substances were probably lost during the thermal processing of acid cannabinoids and the successive steps of concentration-evaporation. These results provide strong arguments in favor of any therapeutic use of an e-liquid containing cannabinoids being subject to a clear legal framework, quality control to ensure consistent product composition, storage recommendations, and an expiry date for consumption.

### 4.4. Manufacture of a BHO Sample

**Figure 4:**
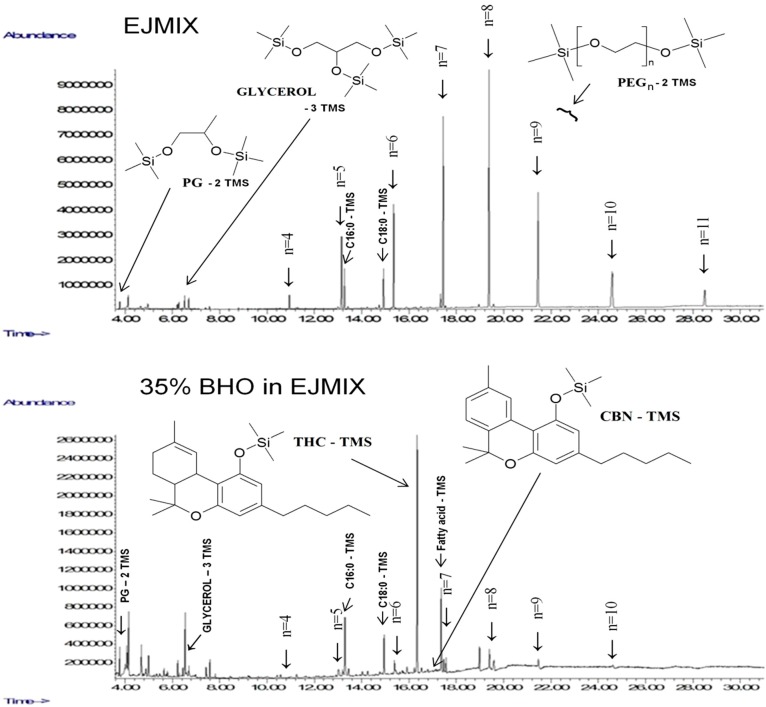
Gas chromatography-mass spectrometry (GC-MS) chromatograms of TMS derivatives of an EJMIX extract and of a BHO/EJMIX 35:65 w/w solution. EJMIX is a commercially available mixture of propylene glycol (PG) and polyethylene glycol (PEG) molecules in unknown proportions (Bloomsdays, USA). The PEG molecules are characterized by their number, n, of “oxyethylene” units. PEG 200 usually contains 4–5 units; PEG 300, 6–7 units; and PEG 400, 8–9 units. PG is propylene glycol or 1,2-propane diol. THC = (−)-*trans*-delta-9-tetrahydrocannabinol; CBN = cannabinol. C16:0 and C18:0 are palmitic and stearic acids, respectively.

Ten grams of medicinal-grade cannabis flower buds (Bedrobinol from Bedrocan BV, The Netherlands) containing 17% total THC (free and acid forms, but mostly THC-A to > 95%) were ground to a coarse powder. This powder, containing 1700 mg total THC, was then placed in a M15 BHO Roller Extractor (15 × 2.8 cm) equipped with a grid and a cellulose filter (Alchimia, Vilamalla (Girona), Spain). Cannabinoids were eluted using three bottles of butane gas (3 × 250 mL/139 g) (Tycoon premium butane gas, Curly & Smooth Handels GmbH, Germering, Germany), yielding a yellow, viscous liquid. The pressure release causes butane ebullition and evaporation. To achieve total evaporation of the butane gas, the extract was warmed in a water bath until the disappearance of the last bubble. Scraping the glass container recovered 1 g of crude BHO extract. This extract was kept at 120 °C for 2 h in a glass container closed with a cap and a septum pierced by a needle to release any pressure increase induced by the formation of carbonic gas. Only 0.7 g of BHO were recovered after decarboxylation. In contrast to the crude extract containing almost exclusively THC-A, only THC with traces of CBN were detected in the heated extract. The heat-treated crude extract was dissolved by sonication in 15 mL ethanol and kept for two days at −20 °C. Following this winterization process, a precipitate and foam were visible in the frozen tube. The brownish, ethanolic phase was cold filtrated in a separation funnel fitted with a glass filter. After evaporation of the solvent (4 h, 50 °C), 680 mg of purified BHO were recovered. One half of the BHO was mixed with two parts of EJMIX e-liquid, while the other half was solubilized in two parts of pure PG solution. The THC concentrations in the BHO were 75% in the EJMIX e-liquid and 64% in the PG e-liquid. The liquids appeared homogenous and no phase separation was observed during one week’s storage at room temperature. With regard to initial and final THC levels, the entire processing of cannabis head buds into BHO extracts was carried out with an extraction yield of about 28%. The GC-MS elution profiles of an EJMIX solution and its corresponding BHO e-liquid, obtained after silylation with MSTFA, are shown in Figure 4. In each chromatographic profile, the various silylated polyethylene glycol (PEG) molecules of increasing chain lengths and ethylene glycol units (from 4 to 11) are clearly visible. In the BHO/EJMIX solution, THC constitutes the main peak. Only traces of CBN were found.

In future studies, these cannabinoid-enriched e-liquids should be vaped in smoking machines designed for tobacco cigarettes in order to evaluate the toxicity of the main-stream aerosol [30]. Health effects as well as effects on behavior and performance could also be assessed in randomized controlled clinical vaping trials.

## 5. Users’ Acceptance and Putative Benefits

### 5.1. Epidemiology

Very few scientific data are available regarding the prevalence of cannabis and e-cig use. On the contrary, a very large number of websites provide instructions on how to prepare cannabis-derived liquids for use in e-cig devices [31]. For example, a Google search (June 2015) of the terms “electronic cigarette” and “cannabis” obtained more than 250,000 results; searching for “electronic cigarette“ and “BHO” obtained 72,000 results; and searching for (“electronic liquid” OR “e-liquid”) AND “marijuana” obtained 420,000 results. The same search, made with Google Scholar, provided a mere 384 results. In December 2013, J.F. Etter, from Institute of Global Health at the University of Geneva’s Faculty of Medicine, in Switzerland, posted a questionnaire on Stop-tabac.ch. This is a website of the Institute of Social and Preventive Medicine (Faculty of Medicine, University of Geneva, Switzerland); “Stop-tabac” provides free, personalized help to people wanting to quit tobacco use. The questionnaire addresses several issues, namely: demographic parameters, habits, feeling effects, beneficial and unwanted side effects for consumers using cannabis in e-cigs or portable electronic vaporizers [32]. Initial results, from a small community of 55 users, suggest that cannabis vaping via e-cigs or e-vaporizers are infrequent behaviors among cannabis users. A few respondents indicated sporadic use of e-cigs. Participants were mainly men, middle-aged, and current users (two days/week) of cannabis e-cigs. The preferred vaped cannabis materials were buds and oil rather than hashish or BHO [33].

### 5.2. Putative Advantages of Cannabis E-Cigarettes over Regular Joints

For regular, heavy adult cannabis smokers, cannabis vaping using e-cigs may present several advantages over smoking typical cannabis joints in terms of harm reduction:

(a) Puffing cannabis vapors generated at low temperatures should be far less toxic than inhaling cannabis smoke formed during combustion in a joint.
(b) Vaping raw cannabis or THC concentrates alone (without tobacco or nicotinic liquids) should diminish the exposure to tobacco toxicants and lessen the addiction to nicotine.
(c) Vaping cannabis using an e-cig, especially by regular or heavy marijuana smokers interested in quitting smoking, could diminish cannabis use and be a first step towards abstinence [34].
(d) Unlike cannabis smoking, vaping is better suited for medical applications [23].
(e) Finally, e-cig technology is rapidly developing, suggesting that more reliable, safer equipment, optimized for the administration of cannabis and other drugs, will be marketed in the near future.

## 6. Health Issues

### 6.1. Acute Toxicity, Risk of Poisoning

Varlet *et al.* [35] tested 42 models of e-liquid refills from 14 brands, concluding that their oral acute toxicity seemed to be of minor concern, based on an ingestion scenario. This was without considering the toxicity of nicotine. However, besides the risks of passive vapor exposure [36], there exist non-negligible dangers of poisoning for children and teenagers via accidental or voluntary ingestion of e-liquids containing appetizing flavors, nicotine, and/or psychoactive drugs [37,38]. Furthermore, these substances go directly into general circulation via the inhalation pathway, and should induce a higher systemic toxicity for having bypassed the first pass hepatic metabolism. Finally, some of these substances are also toxic on contact with skin. Between 2010 and 2014, US poison centers recorded 2405 e-cig exposure calls, most (51.1%) involving toddlers and young children (0–5 years). Most e-cig exposures were reported as inhalations, eye and skin exposures, and there were fewer reported ingestions. The most common adverse health effects recorded in e-cig exposure calls were vomiting, nausea, and eye irritation. One suicide death from intravenous injection of nicotine e-liquid was reported to poison centers [39]. A literature review of research on e-cigs, BHO, and cannabis indicated that scientific investigations assessing the health effects and potential harm caused by BHO vaping were very scarce. Moreover, the pharmacokinetics of cannabinoids after vaping BHO extract in e-liquid remains largely unknown. Loflin and Earleywine (2014) [19] claim that users consider BHO use to be significantly more dangerous than other forms of cannabis use. However, their paper refers to a new method of cannabis inhalation known as dabbing. Dabbing is a special procedure for inhaling THC vapors from butane hash oil in order to feel the effects rapidly: a dab of the wax-like oil is placed on the end of a glass rod that has been heated, using a grill lighter or a blowtorch, so that the BHO concentrate vaporizes immediately.

### 6.2. Chronic Toxicity of Cannabis E-Liquids, Health Issues

Goniewicz *et al.* [40] reported the identity and concentration of 11 toxicants in the aerosols of 12 brands of e-cigs. The levels of toxic and carcinogenic compounds were one to two orders of magnitude lower than in traditional cigarette smoke, but higher than with a nicotine inhaler. The long-term biological effects of nicotine vaping are presently unknown because e-cigs have not been in widespread use long enough for appropriate toxicological investigations [41]. A deviation of the optimum adjustment range of voltage settings, a strong increase in power load, will cause excessive temperatures that may damage the heating coil(s) and induce the formation of toxic pyrolytic by-products and an unpleasant burned taste. With regard to this, Jensen *et al.* (2015) observed that hemiacetals containing formaldehyde can be formed during the e-cig vaping process [42], but only at a relatively high tension voltage (5V). At low voltage (3.3V), and therefore at low coil and vapor temperatures, they did not detect the formation of formaldehyde-releasing agents. The World Health Organization classifies formaldehyde as carcinogenic to humans. Its formation happens when propylene glycol and glycerol are heated in the presence of oxygen to the temperatures reached by commercially available e-cigs operating at relatively high voltage (e.g., as above, 5 volts). However, such high temperatures could make cannabinoid inhalation more efficient because of improved decarboxylation (in case of crude extract vaping) and volatilization rates. The same observation can be drawn for cannabis vaping. The use of e-cigs could, therefore, have very different impacts on the health of users, for example, helping them to moderate their cannabis use or, on the contrary, increase it. The influence of THC on the neurodevelopment of young people [43] and the risk of obesity [44] due to its orexigenic properties should also be considered.

### 6.3. Environmental and Passive Contamination Caused by Cannabis Vaped-Drugs

Passive vaping, also referred to as involuntary vaping, refers to the inhalation of e-cig aerosols by non-smokers and smokers themselves. In forensic sciences, unwanted passive exposure is an important issue because it has been used, regardless of its true relevance or irrelevance, to explain a positive cannabis test result. This argument could change and shift from joint smoking to e-cig vaping. In our opinion, this contamination is less likely: unlike regular tobacco cigarettes, which continuously produce particles from the combustion process, e-cigs emit no side-stream smoke (the smoke released from the tip of a burning cigarette). Furthermore, no vapor is emitted when the e-cig is switched off. The only aerosol released into the air is that exhaled by the smoker (second-hand main-stream vapor). The consumption of e-cigs loaded with nicotinic e-liquid causes emissions of aerosols and volatile organic compounds, such as propylene glycol, glycerol, diacetin, flavoring substances, and nicotine [45]. Schripp and co-workers also found that during the inhalation of e-cig vapor, the size of the aerosol droplets decreases [45]. This effect could result from the deposition and evaporation of the liquid particles in the lung and in the environment after exhalation. Interestingly, the half-life in air of the exhaled vapor stream was found to be very short: only 11 seconds compared to 20 mins for tobacco smoke [46]. The authors conclude that the risk of passive vaping exposure from e-cigs is modest. An accurate evaluation of the overall health risks posed by passive exposure to vaped cannabis or THC aerosols, particularly on children’s health, will require relevant human exposure data, which are lacking at present. Similarly to traditional tobacco smoking, one might then also be contaminated by third-hand vapors deposited on everyday objects (e.g., clothes, furniture, computer keyboards, and remote controls). Vapor pollutants may remain on surfaces and in dust, and may be re-emitted into the gas-phase, or react with other compounds in the environment to form secondary pollutants. Infants and children are more prone to the risks of third-hand smoke exposure than adults [47]. Goniewicz and Lee found that e-cigs are indeed a source of third-hand exposure to nicotine [48]. A further aspect should not be underestimated: children learn by imitating others, especially peers and adults. Frequent passive exposure to e-cigs could therefore significantly increase their desire and urge to vape nicotinic solutions but also to smoke regular cigarettes.

## 7. Discussion and Conclusions

### 7.1. Future Perspectives

The tobacco industry cannot afford to remain inactive in the face of the fast growing market of e-cigs. For instance, one big tobacco company took a share in a start-up developing an innovative product called Ploom^®^ (Japan Tobacco International (JTI), Geneva, Switzerland). The Ploom^®^ is a loose-leaf vaporizer that heats small pods of tobacco, whereas most e-cigs use liquid mixtures of nicotine, fragrance, and glycols. Other companies are also developing similar “potentially reduced-risk products” (e.g., iQos^®^ from Philip Morris Int., Lausanne, Switzerland). Voke^®^ is possibly the world’s first medically-approved “electronic-like cigarette” marketed by a tobacco industry major (Nicoventures Limited, London, Great Britain, a subsidiary wholly owned of British American Tobacco plc, London, Great Britain). The innovative Voke^®^ technology combines an inhaler apparatus with a canister of nicotine, enabling the user to take a dose of nicotine by puffing on a cigarette-shaped stick. Since Voke^®^ uses pressure instead of electrical energy to generate an aerosol for inhalation, no battery or heating element is necessary. By means of direct acquisitions, purchases, and takeovers, the mainstream tobacco industry has also entered the e-cig market [34]. Meanwhile, e-cig and e-liquid technologies developed by small and innovative high-tech firms evolve very quickly. One challenge is to improve the performance and rate of nicotine delivery to the body. With this goal, Pax Labs, Inc. has substituted nicotine salts for free-base nicotine in an e-liquid and has developed new e-cig hardware. The product, called “JUUL”, is claimed to provide a blood nicotine absorption profile approaching that observed after regular tobacco cigarette smoking, unlike most e-cigs currently on the market [49]. Users of cannabis and other psychoactive drugs will, no doubt, take advantage of these developments. Convenient, non-smoked devices enabling systematic delivery, via the lungs, of a variety of substances for therapeutic and “recreational” purposes are eagerly awaited. However, to the best of our knowledge, no equipment currently satisfies all the demands for efficiency (dose precision and accuracy), durability, versatility, robustness, portability, and especially patient and user acceptance. Nevertheless, the administration of cannabinoids using a pen-size vaporizer [50] (or better, with a table vaporizer [10]) currently seems to be the closest means of meeting these multiple demands.

### 7.2. Health Fears, Recommendations, and Concluding Remarks

In summary, the growing popularity of vaping among teenagers could be associated with an increase in nicotine use in this age group. But more than this, the emergence of this “new youth culture of vaping” could weaken the efficiency of anti-smoking campaigns and measures [4]. Thus, a parallel can be drawn with e-cigs used for vaping cannabis extracts, as this technological innovation could attract many young people and thwart cannabis use prevention efforts. Indeed, because cannabis vapors have no suspicious odors (if properly deodorized or flavored), teenagers can use advanced vaping devices without being detected; this is commonly called *stealth vaping* [51]. The use of solutions containing nicotine to prepare e-liquids enriched with THC could also favor a dual addiction to these psychotropic drugs. Adolescence is a time of great sensitivity to environmental factors such as exposure to cannabis, and recent studies have shown that its early, chronic use is correlated with a lower volume of the brain’s grey matter [52], cognitive decline, and performance impairment [53]. These alterations are more obvious when cannabis use started before 18 years old, as suggested by our research group [43] and others [52,53]. Health policy makers must address the development of nicotine and cannabis vaping among adolescents and build specific screening, prevention, and intervention programs in addition to regulation [4]. Regulatory decisions must be taken, and these will face many challenges [54]. The ultimate goal is preventing long-term brain damage and thus guaranteeing good cognitive function for adolescents entering their early adult years. Nevertheless, several issues still need to be examined and clarified before scientifically-based policies and regulations can be put in place specifically targeting cannabis vaping and youth. Future studies should look towards achieving a balance of interests between the possible health-related benefits of vaping and the harm that the substances involved can cause. Observation studies will be needed to define the prevalence of cannabis vaping among adolescents, and experimental studies should assess the short- and long-term toxicity of cannabinoids vaping. Pharmacokinetic and pharmacodynamic investigations are also required. Finally, the health consequences of passive vaping should be also considered.

Although today’s e-cig hardware seems moderately attractive to cannabis users (whether for recreational, abuse, or therapeutic uses), this is likely to change if more efficient, better tolerated, more suitable, and better designed vaporizers and cannabis concentrates are put on the market. However, recognition of these vaporizers by health authorities would be a further sign of encouragement to those promoting cannabis vaping among e-cig enthusiasts, as well as to manufacturers.

In conclusion, the new social phenomenon of vaping may slide from nicotine towards other psychoactive drugs (e.g., THC); it therefore deserves the urgent scientific investigation and strict risk assessments which are especially important when young people are concerned. In particular, the presence of toxic substances in the cannabis aerosols generated by e-cigs—from multiple models, brands, BHO and e-liquid manufacturers—need to be investigated.

## References

[1] Brown C.J., Cheng J.M. (2014). Electronic cigarettes: Product characterisation and design considerations. Tob. Control.

[2] Flahault A., Etter J.F. (2014). Electronic cigarettes: It is urgent to promote them to save lives. Int. J. Public Health.

[3] Earleywine M., Barnwell S.S. (2007). Decreased respiratory symptoms in cannabis users who vaporize. Harm Reduct. J..

[4] Gostin L.O., Glasner A.Y. (2014). E-cigarettes, vaping, and youth. JAMA J. Am. Med. Assoc..

[5] Farsalinos K.E., Polosa R. (2014). Safety evaluation and risk assessment of electronic cigarettes as tobacco cigarette substitutes: A systematic review. Ther. Adv. Drug Saf..

[6] Farsalinos K.E., Spyrou A., Tsimopoulou K., Stefopoulos C., Romagna G., Voudris V. (2014). Nicotine absorption from electronic cigarette use: Comparison between first and new-generation devices. Sci. Rep..

[7] Borrelli F., Fasolino I., Romano B., Capasso R., Maiello F., Coppola D., Orlando P., Battista G., Pagano E., di Marzo V., Izzo A.A. (2013). Beneficial effect of the non-psychotropic plant cannabinoid cannabigerol on experimental inflammatory bowel disease. Biochem. Pharmacol..

[8] Izzo A.A., Borrelli F., Capasso R., di Marzo V., Mechoulam R. (2009). Non-psychotropic plant cannabinoids: New therapeutic opportunities from an ancient herb. Trends Pharmacol. Sci..

[9] Enhanced Delivery of Nicotine, THC, Tobacco, Cannabidiol or Base Alkaloid from an Electronic Cigarette or Other Vapor or Smoke Producing Device Through Use of an Absorption Conditioning Unit US 20140166028 A1. http://www.google.com/patents/US20140166028.

[10] Solowij N., Broyd S.J., van Hell H.H., Hazekamp A. (2014). A protocol for the delivery of cannabidiol (CBD) and combined CBD and 9-tetrahydrocannabinol (THC) by vaporisation. BMC Pharmacol. Toxicol..

[11] Results for: “e-cig”. http://www.hightimes.com/search?search=e-cig,.

[12] “SpaceJuice”, la E-cig Version High. http://www.cannabis.info/fr/abc/10007167-quotspacejuicequot-la-e-cig-version-high.

[13] Game Changer Emulsifying Vegetable Glycerin and BHO for E-Juice. http://skunkpharmresearch.com/game-changer-emulsifying-vegetable-glycerin-and-bho-for-e-juice/.

[14] My Secret to THC E-Cig PG-Liquid. Potent, Effective, Cheap, Easy!!!. http://fuckcombustion.com/threads/my-secret-to-thc-e-cig-pg-liquid-potent-effective-cheap-easy.6287/.

[15] Dussy F.E., Hamberg C., Luginbuhl M., Schwerzmann T., Briellmann T.A. (2005). Isolation of Delta9-THCA-A from hemp and analytical aspects concerning the determination of Delta9-THC in cannabis products. Forensic Sci. Int..

[16] Veress T., Szanto J.I., Leisztner L. (1990). Determination of cannabinoid acids by high-performance liquid chromatography of their neutral derivatives formed by thermal decarboxylation: I. Study of the decarboxylation process in open reactors. J. Chromatogr. A.

[17] Cordoba Garcia R. (2014). The challenge of electronic cigarettes. Aten. Primaria.

[18] McCauley L., Markin C., Hosmer D. (2012). An unexpected consequence of electronic cigarette use. Chest.

[19] Loflin M., Earleywine M. (2014). A new method of cannabis ingestion: The dangers of dabs?. Addict. Behav..

[20] Bloomsday EJMIX. https://www.youtube.com/channel/UC0jqi1VydA8lI-794mhkAKw.

[21] Farsalinos K.E., Romagna G., Tsiapras D., Kyrzopoulos S., Voudris V. (2013). Evaluation of electronic cigarette use (vaping) topography and estimation of liquid consumption: Implications for research protocol standards definition and for public health authorities’ regulation. Int. J. Environ. Res. Public Health.

[22] Freeman D., Dunn G., Murray R.M., Evans N., Lister R., Antley A., Slater M., Godlewska B., Cornish R., Williams J. (2015). How cannabis causes paranoia: Using the intravenous administration of 9-tetrahydrocannabinol (THC) to identify key cognitive mechanisms leading to paranoia. Schizophr. Bull..

[23] Hazekamp A., Ruhaak R., Zuurman L., van Gerven J., Verpoorte R. (2006). Evaluation of a vaporizing device (Volcano) for the pulmonary administration of tetrahydrocannabinol. J. Pharm. Sci..

[24] Creating Your Own Cannabinoid E-Liquid!. http://www.chemsrus.com/forum/10-cannabinoids/43770-creating-your-own-cannabinoid-eliquid.

[25] E-Cigarettes Rigged by Drug Users to Smoke Hallucinogen DMT. http://www.rt.com/uk/220551-vape-dmt-electronic-cigarette/.

[26] Drugs Forum-Experiences-Vaporizer Pen for Crystal Meth Use. https://drugs-forum.com/forum/showthread.php?t=226557.

[27] Robson P.J. (2014). Therapeutic potential of cannabinoid medicines. Drug Test. Anal..

[28] Welty T.E., Luebke A., Gidal B.E. (2014). Cannabidiol: Promise and pitfalls. Epilepsy Curr..

[29] Porter B.E., Jacobson C. (2013). Report of a parent survey of cannabidiol-enriched cannabis use in pediatric treatment-resistant epilepsy. Epilepsy Behav..

[30] Goniewicz M.L., Kuma T., Gawron M., Knysak J., Kosmider L. (2013). Nicotine levels in electronic cigarettes. Nicotine Tob. Res..

[31] Durmowicz E.L. (2014). The impact of electronic cigarettes on the paediatric population. Tob. Control.

[32] Questionnaire for Those Who Use Cannabis in Electronic Cigarettes or Portable Electronic Vaporizers. http://www.stop-dependance.ch/tobacco/ECIG_CAN/index_en.html.

[33] Etter J.F. (2015). Electronic cigarettes and cannabis: An exploratory study. Eur. Addict. Res..

[34] Hajek P., Etter J.F., Benowitz N., Eissenberg T., McRobbie H. (2014). Electronic cigarettes: Review of use, content, safety, effects on smokers and potential for harm and benefit. Addiction.

[35] Varlet V., Farsalinos K., Augsburger M., Thomas A., Etter J.F. (2015). Toxicity assessment of refill liquids for electronic cigarettes. Int. J. Environ. Res. Public Health.

[36] Kassem N.O., Daffa R.M., Liles S., Jackson S.R., Kassem N.O., Younis M.A., Mehta S., Chen M., Jacob P., Carmella S.G. (2014). Children’s exposure to secondhand and third hand smoke carcinogens and toxicants in homes of hookah smokers. Nicotine Tob. Res..

[37] Carstairs S.D., Fujinaka M.K., Keeney G.E., Ly B.T. (2011). Prolonged coma in a child due to hashish ingestion with quantitation of THC metabolites in urine. J. Emerg. Med..

[38] Schipper E.M., de Graaff L.C., Koch B.C., Brkic Z., Wilms E.B., Alsma J., Schuit S.C. (2014). A New Challenge: Suicide Attempt using Nicotine Fillings for Electronic Cigarettes. Br. J. Clin. Pharmacol..

[39] Chatham-Stephens K., Law R., Taylor E., Melstrom P., Bunnell R., Wang B., Apelberg B., Schier J.G., Centers for Disease, Control and Prevention (2014). Notes from the field: Calls to poison centers for exposures to electronic cigarettes—United States, September 2010–February 2014. MMWR.

[40] Goniewicz M.L., Knysak J., Gawron M., Kosmider L., Sobczak A., Kurek J., Prokopowicz A., Jablonska-Czapla M., Rosik-Dulewska C., Havel C. (2014). Levels of selected carcinogens and toxicants in vapour from electronic cigarettes. Tob. Control.

[41] Grana R., Benowitz N., Glantz S.A. (2014). E-cigarettes: A scientific review. Circulation.

[42] Jensen R.P., Luo W., Pankow J.F., Strongin R.M., Peyton D.H. (2015). Hidden formaldehyde in e-cigarette aerosols. N. Engl. J. Med..

[43] Battistella G., Fornari E., Annoni J.M., Chtioui H., Dao K., Fabritius M., Favrat B., Mall J.F., Maeder P., Giroud C. (2014). Long-term effects of cannabis on brain structure. Neuropsychopharmacology.

[44] Dube E., O’Loughlin J., Karp I., Jutras-Aswad D. (2015). Cigarette smoking may modify the association between cannabis use and adiposity in males. Pharmacol. Biochem. Behav..

[45] Schripp T., Markewitz D., Uhde E., Salthammer T. (2013). Does e-cigarette consumption cause passive vaping?. Indoor Air.

[46] Bertholon J.F., Becquemin M.H., Roy M., Roy F., Ledur D., Annesi Maesano I., Dautzenberg B. (2013). Comparison of the aerosol produced by electronic cigarettes with conventional cigarettes and the shisha. Revue Mal. Respir..

[47] Ferrante G., Simoni M., Cibella F., Ferrara F., Liotta G., Malizia V., Corsello G., Viegi G., la Grutta S. (2013). Third-hand smoke exposure and health hazards in children. Monaldi. Arch. Chest. Dis..

[48] Goniewicz M.L., Lee L. (2014). Electronic Cigarettes Are a Source of Thirdhand Exposure to Nicotine. Nicotine Tob. Res..

[49] Vaporization Startup Pax Labs Introduces Juul, Its Next-Gen E-Cigarette. http://techcrunch.com/2015/04/21/pax-juul/#.yvtnbp:eZUX.

[50] Easy to Quit—Medical Marijuana Electric Cigarette-Cannabis E-Cigarette. http://www.easytoquit.org/marijuana-electronic-cigarette.html.

[51] Vaporizer Info-Vaporizing Cannabis. http://vaporizer-info.com/en/cannabis-vaporizer.

[52] Lisdahl K.M., Gilbart E.R., Wright N.E., Shollenbarger S. (2013). Dare to delay? The impacts of adolescent alcohol and marijuana use onset on cognition, brain structure, and function. Front. Psychiatry.

[53] Meier M.H., Caspi A., Ambler A., Harrington H., Houts R., Keefe R.S., McDonald K., Ward A., Poulton R., Moffitt T.E. (2012). Persistent cannabis users show neuropsychological decline from childhood to midlife. Proc. Natl. Acad. Sci. USA.

[54] Benowitz N.L., Goniewicz M.L. (2013). The regulatory challenge of electronic cigarettes. JAMA J. Am. Med. Assoc..

